# Long-term experimental evolution reveals purifying selection on piRNA-mediated control of transposable element expression

**DOI:** 10.1186/s12915-020-00897-y

**Published:** 2020-11-06

**Authors:** Ulfar Bergthorsson, Caroline J. Sheeba, Anke Konrad, Tony Belicard, Toni Beltran, Vaishali Katju, Peter Sarkies

**Affiliations:** 1 Department of Veterinary Integrative Biosciences, Texas A&M University, College Station, TX 77845 USA; 2 MRC London Institute of Medical Sciences, Du Cane Road, London, W12 0NN UK; 3 Institute of Clinical Sciences, Imperial College London, Du Cane Road, London, W12 0NN UK; 4 Present Address: Intituto Gulbenkian de Ciencia, Rua da Quinta Grande, 6, 2780-156 Oeiras, Portugal; 5 Present Address: Centre for Genomic Regulation, PRBB Building, 08003 Barcelona, Spain

**Keywords:** *C. elegans*, Experimental evolution, Transposable elements, Epigenetics, Small RNAs, piRNAs, Chromatin

## Abstract

**Background:**

Transposable elements (TEs) are an almost universal constituent of eukaryotic genomes. In animals, Piwi-interacting small RNAs (piRNAs) and repressive chromatin often play crucial roles in preventing TE transcription and thus restricting TE activity. Nevertheless, TE content varies widely across eukaryotes and the dynamics of TE activity and TE silencing across evolutionary time is poorly understood.

**Results:**

Here, we used experimentally evolved populations of *C. elegans* to study the dynamics of TE expression over 409 generations. The experimental populations were evolved at population sizes of 1, 10 and 100 individuals to manipulate the efficiency of natural selection versus genetic drift. We demonstrate increased TE expression relative to the ancestral population, with the largest increases occurring in the smallest populations. We show that the transcriptional activation of TEs within active regions of the genome is associated with failure of piRNA-mediated silencing, whilst desilenced TEs in repressed chromatin domains retain small RNAs. Additionally, we find that the sequence context of the surrounding region influences the propensity of TEs to lose silencing through failure of small RNA-mediated silencing.

**Conclusions:**

Our results show that natural selection in *C. elegans* is responsible for maintaining low levels of TE expression, and provide new insights into the epigenomic features responsible.

## Background

Transposable elements (TEs) are almost ubiquitous across eukaryotic genomes [1]. Their ability to replicate independently of the host genome, coupled with the existence of multiple copies liable to ectopic recombination means they present a potential threat to genome stability. Moreover, TEs pose a threat to genome function as new integrations can disrupt genes or gene regulatory elements. As a result, organisms have evolved sophisticated control strategies, which protect the genome from TE proliferation. Across eukaryotes, short (20- to 33-nucleotide) RNAs play an important role in the suppression of TE activity. Within animals, Piwi-interacting small RNAs (piRNAs) are paramount in the TE defence armoury [2]. piRNAs are produced from defined genomic loci named piRNA clusters and, after processing, associate with the Piwi subfamily of Argonaute proteins [3]. They recognize TEs through sense-antisense base pairing and target TEs for transcriptional and post-transcriptional silencing [2]. In many model organisms, piRNAs are essential for fertility through their role in controlling TE proliferation in the germline [4].

The nematode *Caenorhabditis elegans* is a well-established model for small-RNA mediated silencing. piRNAs in *C. elegans* are unusual in that the two piRNA clusters on Chromosome IV are composed of individual RNA polymerase II (RNA pol II) transcription loci where each piRNA has its own upstream motif [5–8]. piRNA clusters are located within H3K27me3-rich chromatin, which, together with cis-acting RNA pol II pausing sequences downstream of the piRNA, enforce production of ~ 28 nucleotide piRNA precursors [9]. piRNA precursors are further trimmed to result in mature 21-nucleotide piRNAs with a uracil as the first nucleotide (21 U-RNAs), which associate with the *C. elegans* Piwi protein PRG-1 [5–7]. Downstream of PRG-1, piRNA silencing relies on a nematode-specific class of secondary small RNAs known as 22G-RNAs [6]. 22G-RNA synthesis is carried out by RNA-dependent RNA polymerases using the target RNA as a template, following initiation by piRNA target recognition [10]. 22G-RNAs bind to Argonaute proteins and lead to transcriptional and post-transcriptional silencing of target RNAs [11]. Additionally, 22G-RNAs can be transmitted transgenerationally [12] and as a result, piRNA-initiated silencing can persist for many generations even after piRNAs themselves are removed by mutating PRG-1 [13–15]. Consequently, whilst removal of piRNAs alone has mild effects on TE expression, combining mutations of PRG-1 with mutations disrupting the 22G-RNA biogenesis machinery leads to reactivation of several TEs [16, 17].

Despite the universality of TEs across eukaryotes, there is striking variability both in TE content and TE expression across species. Several interacting factors have been proposed to account for this. First, silencing mechanisms differ between organisms. For example, the entire piRNA pathway has been lost independently multiple times in nematodes [18] and was lost in parasitic flatworms [19, 20] and in dust mites [21]. Second, it is possible that some TEs may have beneficial consequences through their ability to act as reservoirs for evolutionary novelty. For example, up to 60% of human-specific enhancers may be TE-derived [22] and TE insertions have been proposed to substantially rewire the human immune cell transcriptome [23]. TEs themselves may also be co-opted into developmental programs. For example, transcription of L1 RNA is observed at the 2-cell stage in mouse embryogenesis where it may have a direct role in coordinating gene expression programs [24]. Furthermore, TEs may serve as a genome-wide source of regulatory elements [1]. Despite these examples, TEs are overall considered to be detrimental to fitness, and beneficial TE insertions appear overrepresented due to the effects of natural selection in weeding out deleterious insertions [25]. The diversity of TEs across evolution may thus reflect population genetics factors such as population structure and effective population size. For example, even moderately deleterious TE insertions might become fixed in very small populations as the intensity of selection decreases with reduced population size. In agreement with this model, a recent large-scale study across nematodes concluded that genetic drift was likely responsible for differences in TE content across nematodes [26].

In the context of these potential models to explain the diversity in TE content, it is important to understand the extent to which the balance between TE expression and TE regulation is under selection. One way to study this is to use a mutation accumulation (MA) framework in which replicate lines descended from a single common ancestor are propagated under a regime of drastic population bottlenecks for several hundred generations [27, 28]. The maintenance of these lines at a minimal population size attenuates the efficacy of selection, thereby enabling the accumulation of a large, unbiased sample of spontaneous mutations under conditions of genetic drift which can subsequently be identified and their fitness effects investigated.

Previously, MA lines have been used to investigate the rate and spectrum of TE copy-number changes in *Saccharomyces cerevisiae*, *C. elegans* and *Drosophila melanogaster*, thus providing estimates of the rate of TE transposition [29–32]. However, TE expression is not necessarily directly linked to TE copy-number and may have independent fitness consequences. The opportunity to interrogate genome-wide RNA expression has been exploited to investigate the effect of mutations on protein-coding gene expression divergence [33–35]. Here, we use *C. elegans* to extend this approach in order to investigate the effect of spontaneous mutations on TE expression divergence.

The genome of *C. elegans* is approximately 12% repetitive [36]. DNA transposons, which spread via a cut and paste mechanism, are the most abundant [37], with Tc1, the founding member of the Tc1/Mariner family of DNA transposons, particularly well-studied as it is active in laboratory and wild strains [38, 39]. There are also a number of retrotransposons, which spread via a copy-paste mechanism involving an RNA intermediate, of which very few are predicted to be active [40, 41]. Other elements, including Helitrons, are also suggested to have been recently active [42]. Transposable elements are more abundant in repressed regions of the genome that are typically enriched with specific histone modifications such as H3K9me3 [43].

We created spontaneous MA lines of *C. elegans* that were descended from a single worm ancestor and propagated for 409 generations under three population size treatments of *N* = 1, 10 and 100 individuals per generation [44]. The varying population size treatment in the experiment permitted a manipulation of the strength of selection, with the *N* = 1 lines evolving under close to neutral conditions (minimal selection) and an incremental increase in the strength of selection with increasing population size. We employed this framework to investigate how TE expression evolves under conditions of near neutrality and under the influence of increasing selection intensity. We show that overall TE expression increases in MA lines with the smallest population size. We further show that expression increase results in part from failure of piRNA-mediated silencing. Intriguingly, differences in the responses of different TEs to reduced piRNA-mediated silencing depend on the chromatin environment of the TE loci, such that TEs in repressed chromatin domains largely remain silent due to epigenetic memory imparted by 22G-RNAs, whilst in active chromatin domains, increased TE expression is much more likely to occur. Together, our results demonstrate for the first time that robust control of TE expression is under selection in animals. Importantly further, our results provide new insight into how the chromatin environment interacts with piRNA-mediated silencing to control TE expression.

## Results

### Relaxed selection leads to increased TE expression

**Fig. 1:**
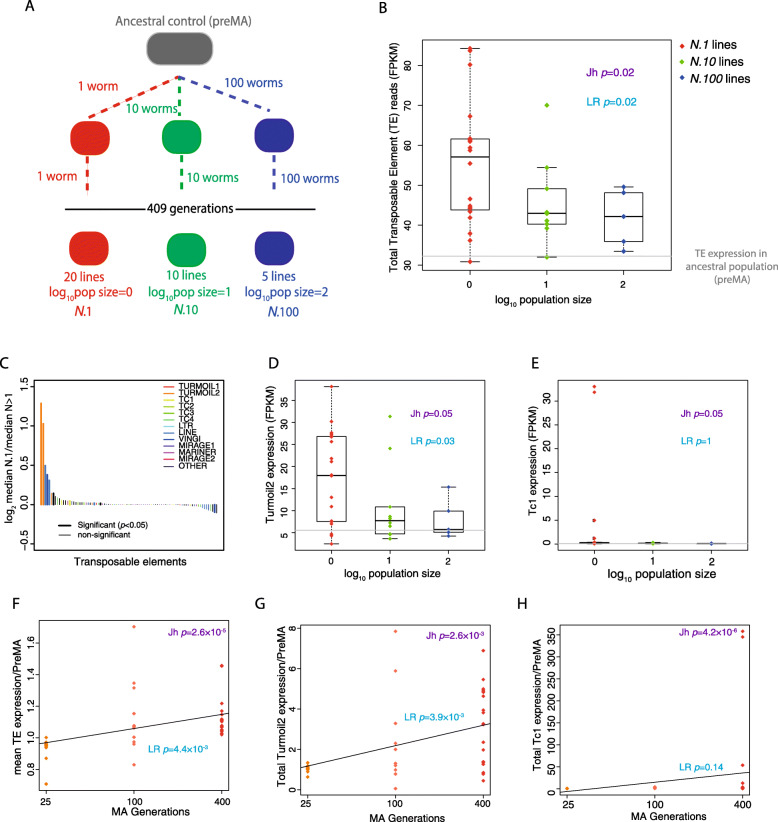
Increased expression of transposable elements in mutation accumulation lines. **a** Diagram of the mutation accumulation (MA) experimental design with different population sizes. **b** Overall transposable element expression in MA lines as a function of population size. Boxplots in all figures presented show the median and the interquartile range with the error bars 1.5 times the interquartile range. Here and in all other figures, the corresponding value for the ancestral control (pre-MA) is marked by a grey line. **c** Expression change in individual TEs, as defined by the fold-change between *N*.1 lines versus *N*.10 and *N*.100 lines combined (*N* > 1) coloured according to family. Statistically significant changes according to Wilcoxon unpaired test *p* < 0.05 are illustrated in bold colours. **d** Expression changes in Turmoil2 elements. **e** Expression changes in Tc1 elements. **f** Total TE expression after different numbers of generations of mutation accumulation in lines with a population size of 1. **g** Turmoil2 expression after different numbers of generations of mutation accumulation with a population size of 1. **h** Tc1 expression after different numbers of generations of mutation accumulation with a population size of 1

In order to assess the effect of selection on TE expression, we generated spontaneous MA lines of *C. elegans* and propagated them by randomly selecting *N* individuals at each generation, where *N* was either 1, 10 or 100 [44]. Henceforth, we refer to the three population size treatments as *N*.1, *N*.10 or *N*.100 (Fig. 1a). We propagated the lines for 409 generations; we isolated RNA and performed RNA sequencing to investigate TE expression. The fundamental difference between these treatments is the size of the bottleneck that the population is subjected to in each generation. However, it is inevitable that the population density will also vary between the three population size treatments, resulting in environmental differences that could introduce variation in gene expression amongst the experimental lines maintained at these three population sizes. To counteract this, all lines were maintained in similar conditions including no population bottleneck differences for three generations prior to RNA extraction. Therefore, the most likely explanation for any differences in TE expression that we observe is the different strength of purifying selection between the three population size treatments during MA. It should be noted that although epigenetic effects affecting endogenous genes lasting longer than three generations are very rare [45], we cannot formally exclude them as a possibility.

MA lines from all three population size treatments showed an increase in total TE expression relative to the pre-MA ancestral control; moreover, *N*.1 had higher total TE expression than *N*.10 or *N*.100. Across increasing population sizes, we observed a monotonic decrease in total TE expression (using the non-parametric Jonckheere test for ordered medians with groupings of *N*.1, *N*.10 and *N*.100 in that order, henceforth Jh, *p* = 0.02; Fig. 1b). Similarly, linear regression analysis showed a significant negative relationship between increasing population size and TE expression (linear regression; henceforth LR, *p* = 0.02; Fig. 1b). The mean expression across all changes in TEs normalized to the pre-MA ancestral control showed a significant tendency to decrease as the population size increased (Jh, *p* = 0.003; LR, *p* = 0.02; Additional File 1: Fig. S1A).

Protein-coding gene expression diverges during MA in a variety of model organisms including *C. elegans* [33, 34, 46]. In addition to an overall increase, TE expression also appears to show broader distributions in the *N*.1 lines compared to the *N*.10 and *N*.100 lines (Additional File 1: Fig. S1B). To test this directly, we estimated the variation in the expression of each individual TE and each individual gene. To control for potential changes in the mean expression, which can affect noise, we calculated the Fano factor [var(x)/mean(x)] within *N*.1, *N*.10 and *N*.100 lines separately. Fano factors for TEs and genes were higher in *N*.1 lines relative to *N*.10 or *N*.100 lines (TEs: Wilcoxon paired test, *p* = 0.015 and 0.004 for *N*.10 and *N*.100; genes: Wilcoxon paired test, *p* < 1 × 10^−16^ for both *N*.10 and *N*.100; Additional File 1 Fig. S1C, S1D). To control for the possibility that the larger number of *N*.1 lines might lead to higher variance, we calculated TE Fano factors from 1000 subsets of five *N*.1 lines and all 252 subsets of five *N*.10 lines and compared these to the five *N*.100 lines. This showed the same trend as the full dataset (Additional File 1: Fig. S1E). To further investigate the variation in expression, we calculated the total variance in the change in expression of all TEs or all genes between each line and the pre-MA ancestral control. Variance in the differences in both TE and gene expression increased with smaller population sizes (Jh, *p* = 0.008 and 0.009, respectively) (Additional File 1: Figs. S1F, S1G). However, importantly, there was no correlation in overall variance between TEs and genes in the same line (Additional File 1: Fig. S1H), showing that TE expression and gene expression diverge independently.

In order to understand loss of repression of TE expression in more detail, we investigated the expression changes of different families of TEs. We detected 87 TEs for which we obtained robust RNA-Seq coverage in at least one line. We used RepeatMasker to classify these TEs into different families (Additional File 1: Fig. S2A, S2B). We detected expression from several major families of TEs (Additional File 1: Fig. S2A, SB), spread across all chromosomes (Additional File 1:Fig. S2C). Our set included TEs in several different chromatin environments and those targeted by small RNAs (Additional File 1: Fig. S2D, S2E). We investigated the change in TE expression between *N*.1 and *N* > 1 lines (combined *N*.10 and *N*.100 lines) and tested for monotonic median increase with decreasing population size. As expected, the majority of TEs that showed a significant difference between *N*.1 and *N* > 1 lines had increased expression in *N*.1 compared to the other lines (Fig. 1c). However, individual TEs displayed different patterns of expression change. Some TEs, notably the DNA transposon Turmoil2, showed more consistent increases across the *N*.1 lines compared to the *N*.10 and *N*.100 lines (Fig. 1d). Indeed, the majority of the total effect on TE expression seen in Fig. 1b could be attributed to one TE family, the Turmoil2 TEs, which showed a large expression increase across the majority of the *N*.1 lines (Fig. 1c, d). Contrastingly, some TEs, notably the Mariner family DNA transposon Tc1, showed a burst-like pattern of expression where a large increase in expression was observed in a few *N*.1 lines whilst retaining low expression in the remaining *N*.1 lines as well as the *N* > 1 lines (Fig. 1e).

We next investigated the time course of TE desilencing during propagation of the MA lines. We performed gene expression analysis by RNA-Seq on 11 *N*.1 lines at 25 and 100 MA generations. Median relative TE expression showed a significant increase with increasing numbers of MA generations (Jh, *p* = 2.6 × 10^−5^; Fig. 1f). Linear regression analysis confirmed a positive relationship between increased numbers of MA generations and increased TE expression (LR, *p* = 4.4 × 10^−3^; Fig. 1f). Different TEs showed different kinetics of desilencing. Turmoil2 showed a positive relationship between the number of MA generations and expression (LR, *p* = 3.9 × 10^−3^) and a monotonic increase in median expression (Jh, *p* = 2.6 × 10^−3^; Fig. 1g). Contrastingly, Tc1 desilencing did not show a positive linear relationship between the number of MA generations and expression (LR, *p* = 0.14; Fig. 1h) though there was a significant increase in median expression (Jh, *p* = 4.2 × 10^−6^).

**Fig. 2:**
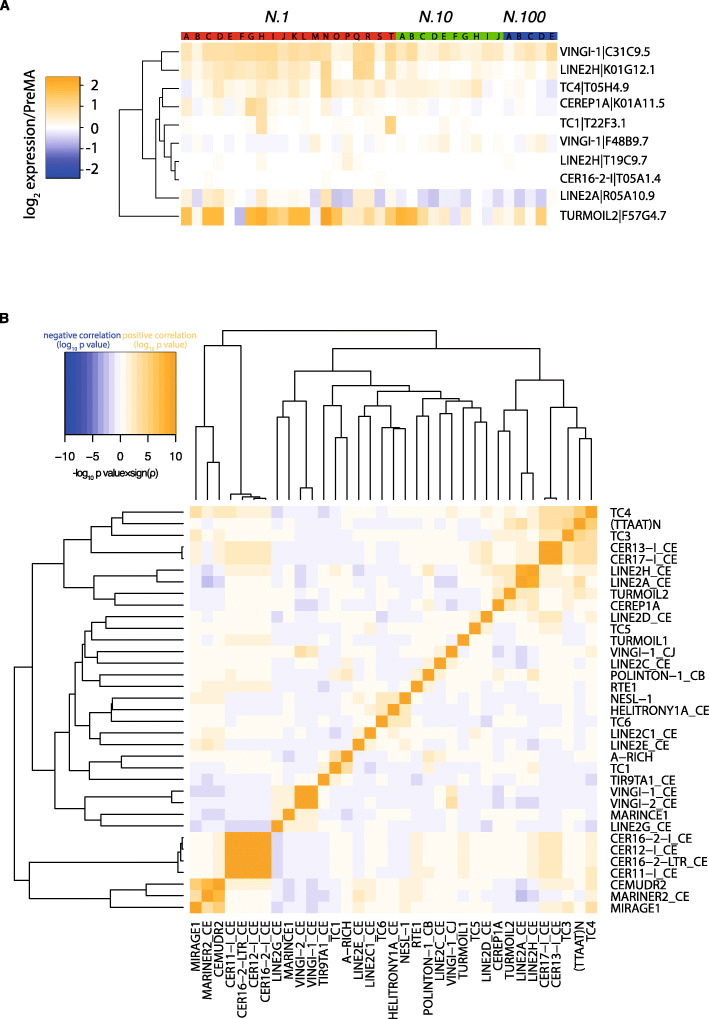
**a** Heatmap of expression of individual TE elements across different lines. TEs with significant differences in expression between different population size treatments are shown. **b** Heatmap illustrating the significance in correlation in expression between different TEs across all the MA lines. The colour intensity shows the significance of Spearman’s rank correlation coefficient with blue showing a negative correlation and orange a positive correlation

We further investigated whether the expression of TEs in individual MA lines correlated with the expression of other TEs. The majority of TEs showed little correlation with the expression of other TEs (Fig. 2a, b). The TEs with the most statistically significant increases in expression, Turmoil2 and certain non-LTR retrotransposons of the LINE2 family (Fig. 1c) clustered together (Fig. 2b). However, not all LINE2 elements clustered similarly, thereby making the significance of this observation unclear.

**Fig. 3:**
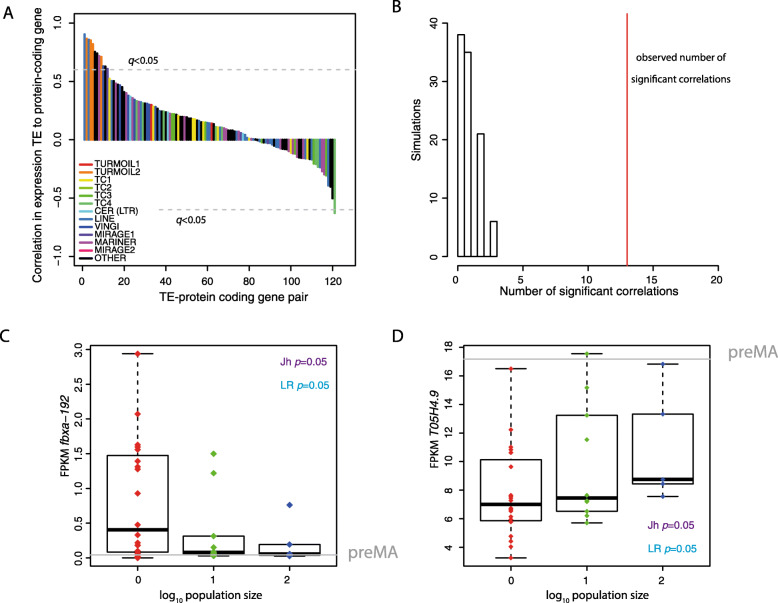
Expression of protein-coding genes near to TEs. **a** Rank correlation coefficient across pairs of TEs and protein-coding genes within 2 kb. **b** comparison between the number of statistically significant pairs observed in the analysis performed in **a** with the random expectation based on 100 samples replacing each protein-coding gene in the TE-protein-coding gene pair with a random gene more than 2 kb away from the TE. **c** Example of a protein-coding gene (*fbxa-192*) close to a TE, which exhibited increased expression in *N*.1 lines along with the TE. **d** Example of a protein-coding gene (T05H4.9), which exhibited decreased expression in N.1 lines contrasting to the nearby TE

We next investigated whether changes in expression of specific TEs was associated with changes in expression of nearby protein-coding genes. We identified protein-coding genes within 2 kb of transposable elements and tested for a correlation between expression changes in the TEs and nearby protein-coding genes. Nine TEs showed statistically significant correlation (Benjamini-Hochberg adjusted *p* value *q* < 0.05) to the expression of 13 adjacent protein-coding genes (Fig. 3a), a statistically significant enrichment over what would be expected by chance (Fig. 3b; empirical *p* < 0.01, 100 simulations; see the ‘Methods’ section). As an example, the gene *fbxa-192*, located in close proximity to a Turmoil2 element, showed a significant increase in expression as population size decreased (Jh, *p* = 0.05; LR, *p* = 0.5; Fig. 3c). Interestingly, we also identified one example with a significantly negative correlation. Indeed, the nearby gene T05H4.7 showed increasing expression with increasing population size (Fig. 3d; Jh, *p* = 0.04; LR, *p* = 0.05) in contrast to the nearby TE. This gene is a predicted chitinase, potentially important in immunity to oomycete infection [47]. Together these analyses demonstrate that changes in TE expression under conditions of reduced selection are sometimes associated with nearby gene expression changes.

### Expression of TEs is weakly associated with increased copy-number

**Fig. 4:**
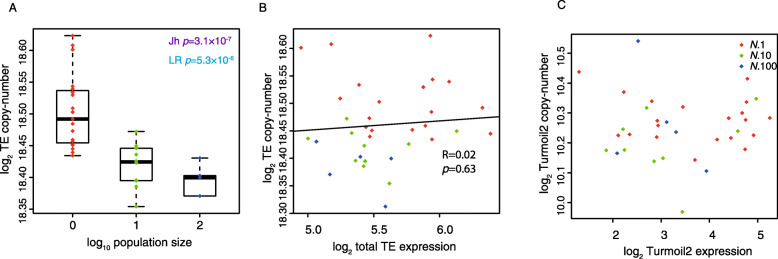
Relationship between TE copy-number and expression in MA lines. **a** Increase in TE copy-number is associated with stronger genetic drift (weaker selection). Data shows mean of TE copy-number across all TEs normalized to the mean across all lines. **b** Correlation between total change in TE copy-number across all TEs and total TE expression. **c** Lack of correlation between Turmoil2 RNA levels and copy-number changes in MA lines

TEs are capable of replicating independently of the host genome and thus their copy-number might change across MA lines. We sequenced the genomes of the MA lines after 409 generations and mapped the reads to consensus TE sequences thereby obtaining estimates of copy-number variation (CNV) for each TE family. Median TE copy-number increased with decreasing population size (Jh, *p* = 8.0 × 10^−4^; LR, *p* = 4.2 × 10^−3^ Fig. 4a). Within individual lines, there was no significant correlation between change in total TE expression and total TE copy-number (Fig. 4b). Furthermore, Turmoil2 elements, which exhibited the largest changes in expression, displayed no correlation between expression changes and copy-number increases (Fig. 4c). We conclude that increased expression of TEs is not always directly coupled to increased TE copy-number.

### Alterations in small RNA levels are associated with TE expression changes

**Fig. 5:**
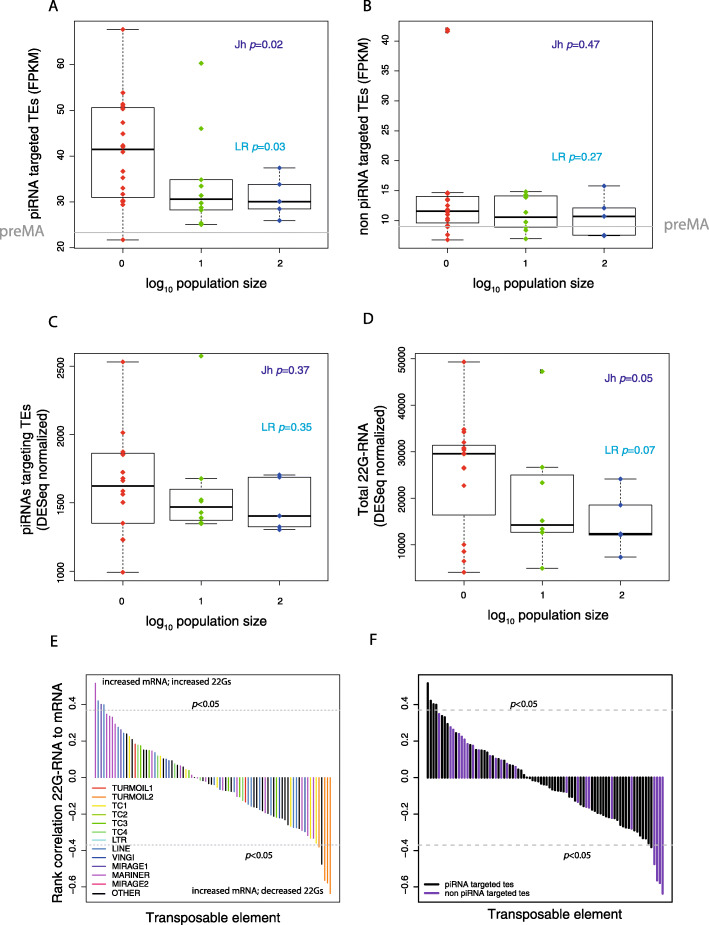
Perturbed 22G-RNAs are associated with changes in TE expression in MA lines. **a**, **b** Comparison of differences in TE expression in MA lines of different population sizes for piRNA targeted and non-piRNA targeted TEs, respectively. See Additional File 1: Fig. S2 for the number of TEs targeted by piRNAs. **c** Total piRNA levels in MA lines across different population sizes. **d** Levels of 22G-RNAs mapping to all TEs in MA lines at different population sizes. **e** Barplot illustrating Spearman’s rank correlation coefficient between change in TE expression and the levels of 22G-RNAs mapping to the same TE, coloured by the identity of the TE. **f** As in **e**, but coloured according to whether the TE is targeted by piRNAs or not

We investigated whether changes in regulation of TE expression could explain the loss of silencing observed during mutation accumulation. In *C. elegans*, piRNAs and 22G-RNAs are important small RNA classes involved in TE silencing [6, 16, 17, 48]. To test whether piRNAs are important in the loss of silencing of TEs in the *N*.1 lines, we remapped recently published cross-linking immunoprecipitation (CLIP) data [49] to identify TE transcripts that are bound by piRNA-Piwi complexes in vivo. Approximately 25% of TE transcripts with RNA-Seq reads were targeted by piRNAs. TEs targeted by piRNAs showed a statistically significant increase in total expression in the *N*.1 lines (Jh, *p* = 0.02; LR, *p* = 0.03; Fig. 5a) whilst TEs that were not targeted by piRNAs were not significantly altered (Jh, *p* = 0.47; LR, *p* = 0.27; Fig. 5b).

We tested whether defective piRNA-mediated silencing might account for increased expression of TEs targeted by piRNAs. piRNAs in *C. elegans* are expressed from individual promoters as a result of RNA polymerase II transcription [50–52]. We considered two potential mechanisms that might give rise to altered piRNAs. One possibility is that mutations in the piRNA sequences might occur in individual lines, which might affect their ability to recognize transposable elements and thus interfere with TE silencing. There was no significant trend for *N*.1 lines to have more mutations in piRNAs than *N*.10 or *N*.100 lines (Jh, *p* = 0.12; Additional File 1: Fig. S3A). Moreover, only an average of 1.1 DESeq-normalized reads per line were detected from mismatches in piRNA sequences across the *N*.1 lines. Thus, we conclude that the increase in TE expression is unlikely to be related to mutations in specific piRNAs.

We next considered the expression of individual piRNA loci. Total piRNA levels and levels of piRNAs targeted to TEs were not significantly altered across different population sizes (Fig. 5c; Additional File 1: Fig. S3B). Thus, changes in piRNA expression are unlikely to explain the changes we observed in TE expression. 22G-RNAs act downstream of piRNAs to bring about target silencing [6]. Surprisingly, although 22G-RNAs silence TEs, we found that the total levels of 22G-RNAs mapping to TEs were increased in lines maintained at smaller population sizes, although this increase was only marginally significant (Jh, *p* = 0.05; LR, *p* = 0.07; Fig. 5d). 22G-RNA levels mapping to TEs targeted by piRNAs behaved similarly (Additional File 1: Fig. S3C). To examine this in more detail, we analysed 22G-RNAs at individual TEs. We investigated the correlation between TE expression and 22G-RNA levels across all the different lines, using Spearman’s rank correlation coefficient to avoid making the assumption of linearity (Fig. 5d). An approximately equal number of TEs demonstrated positive correlations (22G-RNA levels increased and expression also increased) and negative correlation coefficients (22G-RNA levels decreased with expression increased; Fig. 5e). This suggests that at some TE loci, 22G-RNA levels increase in response to increased TE expression, whilst in others, reduced 22G-RNA levels are associated with increased TE expression. Turmoil2 elements were particularly prominent amongst TEs displaying increased expression and reduced 22G-RNA levels (Fig. 5e). Consistently, total Turmoil2 22G-RNAs showed a negative correlation with changes in 22G-RNA levels relative to the parent line (LR, *p* = 2.0 × 10^−5^; Additional File 1: Fig. S3D). Notably, four out of the five TEs with clear negative correlation between expression and 22G-RNA levels were piRNA targets (Fig. 5f), a statistically significant enrichment relative to all TEs (Fisher’s exact test, *p* = 0.035). This suggests that loss of piRNA targeting might be responsible for increased expression of some TEs due to reduced levels of 22G-RNAs. 22G-RNAs induced downstream of piRNAs bind to HRDE-1 and WAGO-1 and consistently, HRDE-1 and WAGO-1 targets identified on the basis of immunoprecipitation experiments [14, 53] included TEs with increased expression and reduced 22G-RNA levels (Additional File 1: Fig. S3E, S3F). Furthermore, both WAGO-1 and HRDE-1-targeted TEs showed increased expression as population size decreased (Additional File 1: Fig. S3G, SH).

### Chromatin environment is associated with the relationship between 22G-RNA levels and TE expression

22G-RNAs interact with chromatin-modifying factors to control expression of TEs [43]. piRNA-mediated silencing has been directly linked to the generation of H3K9me2/3-marked nucleosomes (“classical heterochromatin”), and this has been proposed to be important for transcriptional silencing induced by piRNAs [13, 14, 43, 54]. Additionally, it is becoming clear that a large proportion of the autosomal DNA in *C. elegans* can be divided into two categories. “Active” domains, containing H3K36me3, contain genes that are expressed in the germline and broadly expressed across somatic tissue. “Regulated” domains hold H3K27me3-marked nucleosomes and contain genes that are silent in the germline with restricted expression in specific tissues or developmental stages [55–57]. These domains are largely stable through development, including in the adult germline [55, 58]. These chromatin domains are not found on the X chromosome, which has instead an alternative configuration of chromatin enriched in H4K20 mono-methylation and H3K27 methylation due to the activity of the dosage compensation complex [59]. We examined the influence of these types of chromatin on the response of TEs to mutation accumulation.

**Fig. 6:**
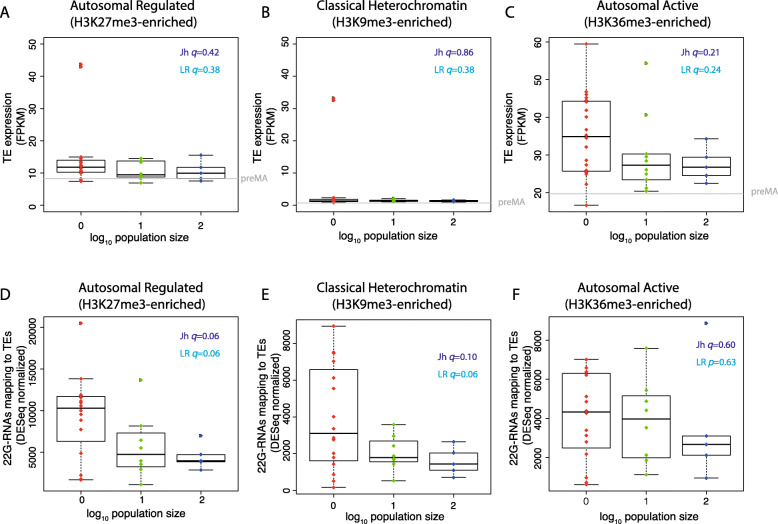
Chromatin environment controls alterations in small RNA-mediated silencing in MA lines. **a**–**c** Expression changes in all TEs in regulated (H3K27me3), classical heterochromatin (H3K9me2) and active (H3K36me3) chromatin domains. *p* values are Wilcoxon unpaired test. See Additional File 1: Fig. S2 for the number of TEs in each domain. **d–f** Changes in 22G RNAs mapping to all TEs in regulated, classic heterochromatin and active domains

TEs on the X chromosome showed no difference in expression between *N*.1 lines and *N*.100 lines (Additional File 1: Fig. S4A, S4B). There was also no significant difference in TE expression across population size in autosomal regulated domains, classical heterochromatin or active domains (Benjamini-Hochberg corrected *p* value *q* > 0.1; Fig. 6a–c). The rare examples of *N*.1 lines showing significantly higher expression of TEs in classical heterochromatin corresponded to lines in which Tc1 reactivation occurred, consistent with enrichment of Tc1 elements within these regions [43]. However, TEs in active domains had significantly higher expression in the *N*.1 than all *N* > 1 lines together (Wilcoxon unpaired test, *p* = 0.02).

We next examined 22G-RNAs mapping to TEs across different chromatin domains. TEs in H3K27me3-enriched autosomal regulated domains showed significantly (Benjamini-Hochberg corrected *p* value *q* < 0.1) increased levels of 22G-RNAs as population size decreased (Jh Benjamini-Hochberg adjusted *p* value *q* = 0.06; LR, *q* = 0.06). TEs in classical heterochromatin also showed significantly increased levels (Jh, *q* = 0.06; LR, *q =* 0.10). However, in active domains, there was no significant change in 22G-RNA levels (Fig. 6d–f). Thus, increased 22G-RNAs occur predominantly in TEs within silent autosomal chromatin, marked by either H3K27me3 or H3K9me3.

### AT-rich sequences in TEs reduce the generation of 22G-RNAs

**Fig. 7:**
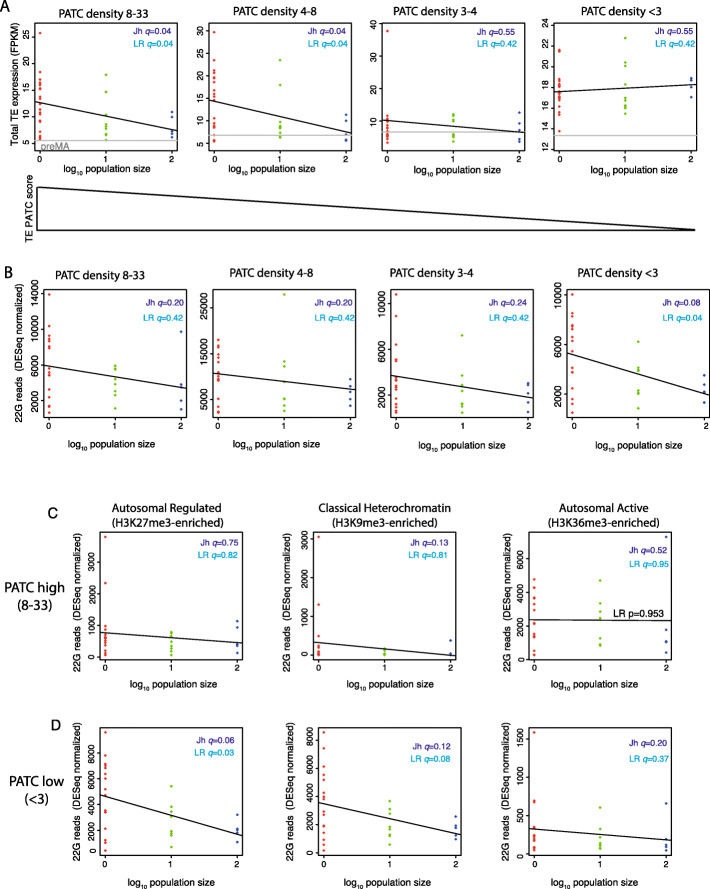
PATC content influences 22G-RNA generation in MA lines. **a** Expression differences in TEs in four equal-size bins of 21 TEs with decreasing PATC content. **b** 22G-RNAs across the bins used in **a**. **c**, **d** Stratification of bins from **a** and **b** into regulated (H3K27me3) chromatin, active (H3K36me3) chromatin and heterochromatin (H3K9me2) domains. High PATC is the top bin and low PATC is the lowest bin

A recent study has demonstrated that silencing of both transgenes and endogenous genes by 22G-RNAs is inhibited by a high content of periodic repeats of AT-rich sequences, known as PATCs [60]. We tested how PATC density within TEs was associated with changes in their expression under reduced selection. High PATC density corresponded to significant (Benjamini-Hochberg adjusted *p* value *q* < 0.1) reactivation of TEs (Jh, *q* = 0.04; LR, *q* = 0.04) whereas TEs with low PATC density did not show an increase in expression (Fig. 7a). Contrastingly, only TEs with low PATC density showed significantly increased 22G-RNAs in *N*.1 lines relative to *N*.10 and *N*.100 (Jh, *q* = 0.08; LR, *q* = 0.04; Fig. 7b). Importantly, this effect was specific to PATC sequences as GC-content alone had no significant effect on either TE expression or small RNA generation (Additional File 1: Fig. S5A, S5B). We conclude that low PATC density is required for 22G-RNA generation, which may be required to restrain TE activation. We tested whether the chromatin environment modulated the effect of PATC sequences on TE reactivation in MA lines. Importantly, PATC content was similar in TEs across active, classic heterochromatin and regulated domains (Additional File 1: Fig. S5C). 22G-RNAs were significantly increased in low PATC regions within regulated domains (Jh, *q* = 0.06; LR, *q* = 0.03) and classical heterochromatin domains (Jh, *q* = 0.12; LR, *q* = 0.08) but not in active domains (Fig. 7c, d). In contrast, there were no significant changes in 22G-RNA levels in high PATC regions within these domains (Fig. 7c, d). Thus, the generation of increased 22G-RNAs against TEs in MA lines is associated with both low PATC content and autosomal H3K27me3 “regulated” and classical H3K9me3-enriched heterochromatin environments.

## Discussion

Our analysis of how the interplay between TE expression and TE silencing factors changes over 409 generations at small population sizes provides the first clear view of how TE expression diverges under reduced selection in animals. Additionally, a closer analysis of how TE control mechanisms are affected in the MA lines offers new insight into the fundamental mechanisms of TE silencing in *C. elegans*, underlining the ability of experimental evolutionary studies to derive fundamental molecular insights. Here, we discuss each of these aspects of our work in turn.

### The effect of selection on TE expression

Here, we demonstrate that in *C. elegans*, TEs drift to higher expression under conditions of strong genetic drift and reduced efficiency of selection. This is consistent with the action of purifying selection on TE expression. Only a single TE, Vingi-1, showed the opposite trend of lower expression in *N*.1 lines relative to the *N*.10 or *N*.100 lines (Fig. 1b). However, the expression of this element was actually lower in the ancestral control than in the *N*.10 or *N*.100 lines. Thus, the significance of this observation is unclear. Our observations suggest that the expression of most TEs is largely detrimental and TE expression is under purifying selection. It remains a formal possibility that TE activation is beneficial under fluctuating environmental conditions or low fitness as opposed to the stable environment of the laboratory. It is worth noting that we found a significant tendency for nearby protein-coding genes to be both up- and downregulated in association with changes in TE expression, which in turn may contribute to phenotypic variation of importance under certain environmental conditions.

An important point raised by our results is that not all expression increases of TEs are linked to increasing copy-number; indeed, many lines with very high expression of specific TEs display no evidence of increased copy-number. This suggests that many TEs replicate inefficiently in *C. elegans* such that even very large increases in expression levels do not automatically result in increased copy-number. This also implies that TE expression may be detrimental without directly posing a threat to genome integrity, potentially through effects on endogenous gene expression networks or through toxicity of repetitive RNA within the cell [61].

Phenotypic analyses of previous *C. elegans* MA experiments suggest that the decline in fitness in *N* = 1 lines results primarily from a few mutations with large effects [44, 62–65]. Similar results have been obtained in experimental evolution studies in *D. melanogaster* [66] and bacteria [67, 68]. In our study, purifying selection at larger population sizes (*N*.10 and *N*.100) would eliminate such large-effect mutations and indeed, our *N*.10 and N.100 lines exhibited no evidence of fitness reduction over the course of successive bottlenecking for 409 generations [44, 62]. Analysis of gene expression data from MA experiments from *C. elegans*, *D. melanogaster* and *S. cerevisiae* concluded that large effect mutations are also responsible for changes in protein-coding gene expression [46]. However, our results for TE expression do not seem to fit with this model because overall TE expression, which is largely driven by Turmoil2 and non-LTR elements (see Fig. 1), increases gradually with time across the *N*.1 lines and is also increased, although less so, in the *N*.10 and *N*.100 lines. The expression of Tc1 is an exception to the overall trend as it is not affected in *N*.10 or *N*.100 lines but a small number of *N*.1 lines show markedly increased Tc1 expression. Thus, Tc1 reactivation may be dominated by a few mutations with large effect. This difference might be related to the different mechanism of silencing of Tc1 compared to Turmoil2 elements as discussed further below.

### Weakened piRNA silencing is responsible for increased expression of TEs under relaxed selection

Our investigations of the molecular mechanisms behind reduced silencing of TEs in MA lines strongly suggest that defective piRNA silencing is a major culprit. Only piRNA-targeted TEs show significantly increased expression in MA lines, and, whilst piRNAs themselves do not seem to change significantly in MA lines, the levels of 22G-RNAs that act as effectors of piRNA silencing are perturbed at the Turmoil2 TEs that show increased expression. Why is piRNA silencing so vulnerable to mutation accumulation? TE silencing and activation in organisms are likely in a precarious equilibrium due to a constant evolutionary arms race between TEs and their host genome. As a result, many mutations could converge on the piRNA pathway to throw TE silencing out of balance.

### New insights into the role of chromatin in the piRNA pathway

**Fig. 8:**
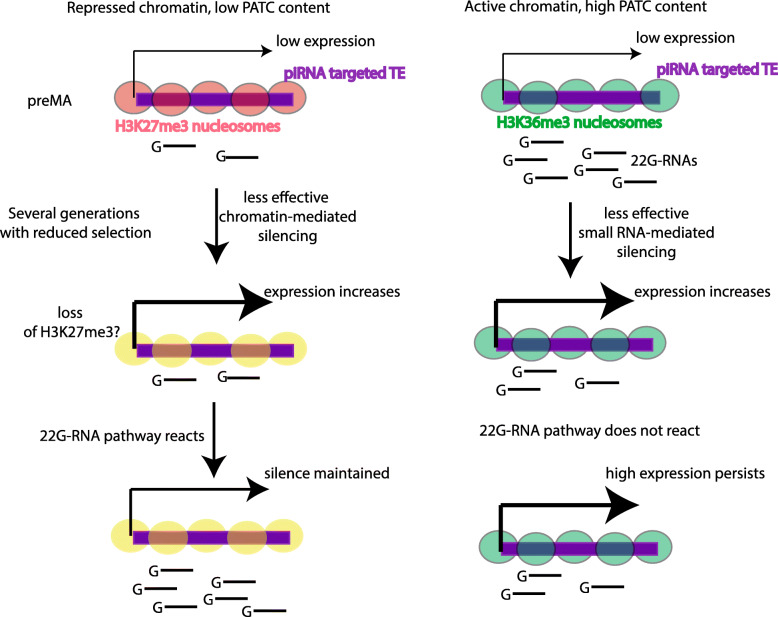
Chromatin and PATC together influence TE control in MA lines. Model for how chromatin environment contributes to TE desilencing in MA lines. The model illustrates two simplified scenarios consistent with our results. On the left, a TE (purple line) in an autosomal regulated domain, enriched in H3K27me3 marked nucleosomes (pink), is illustrated. Continued propagation under relaxed selection leads to loss of chromatin-mediated silencing, potentially accompanied by a loss of H3K27me3 nucleosomes (yellow). However, the 22G-RNA pathway reacts to the increased expression to resilence the TE so that the expression of the TE does not increase. The right-hand side shows the contrasting scenario that takes place at a TE locus (purple) within an active domain marked by H3K36me3-enriched chromatin (green). Here, relaxed selection affects piRNA-mediated 22G-RNA generation directly, leading to loss of silencing and the expression of high levels of TEs from this region

Our analysis of how mutation accumulation affects TE silencing provides novel insights into how the chromatin environment of TEs might affect piRNA-mediated silencing in *C. elegans* (Fig. 8). TEs in autosomal regulated domains, enriched in H3K27me3-marked nucleosomes, and in classical heterochromatin marked by H3K9me3, are less prone to reactivation than those in active chromatin regions. Mechanistically, we propose that this is because 22G-RNA levels in repressive chromatin regions are stable or even increased, whilst 22G-RNA levels mapping to TEs in active regions are reduced in MA lines with increased expression. This result may also explain why reactivation of Tc1 elements occurs less frequently than Turmoil2 elements, because Tc1 elements are predominantly located in repressed domains and are therefore silenced more robustly.

What is the mechanism whereby silencing memory is supported in repressed chromatin regions? The simplest possibility is that generation of 22G-RNAs is directly promoted by repressive chromatin modifications. In line with this possibility, a mutually reinforcing loop between H3K9 methylation and small RNAs is well documented in fission yeast [69], and H3K9 methylation factors contribute to silencing of transgenes in *C. elegans* [13, 14, 43, 54] although the situation is more complicated for endogenous genes [70]. Our observations hold equally well for autosomal H3K27me3-repressed chromatin. These observations are consistent with a previous study reporting that piRNA targeting was correlated with increased H3K27me3 levels mediated by the nuclear silencing factor NRDE2 [71]. We propose that the nuclear small RNA pathway responds differently depending on whether surrounding genes are active or repressed to detect and quell aberrant gene activation. This model will be of interest for further mechanistic investigation of small-RNA mediated silencing in *C. elegans*.

## Conclusions

The control of the expression of specific TEs is vulnerable to the effects of mutation, resulting in increased expression over 409 generations of growth under minimal population size. The vulnerability of TEs to reactivation depends their genomic location and silencing mechanism, as well as the sequence properties of the TE. Our research acts as a key starting point to understand the balance between selection, genetic drift in shaping TE diversity within and across species.

## Methods

### Spontaneous mutation accumulation (MA) experiment at varying population sizes and its theoretical underpinnings

The descendants of a single wild-type Bristol (N2) hermaphrodite were used to establish 35 MA lines. Twenty MA lines were propagated through single individual descent, whilst ten lines were bottlenecked to ten individuals, and five lines were bottlenecked to 100 individuals every generation [44, 62, 72]. The size of these bottlenecks was ensured through careful counting of randomly chosen L4 larvae selected to establish a new generation every 4 days. All populations were grown on NGM plates (Nematode Growth Medium) at 20 °C (Fig. 1a). The lines were propagated through 409 generations or until extinction under standard laboratory conditions. Populations of size *N* = 1 (*N*.1 lines) and 10 (*N*.10 lines) were maintained on 60 × 15 mm petri dishes seeded with 250 μl of *Escherichia coli* (OP50) in YT medium. *N* = 100 (or *N*.100) populations were housed on 90 × 15 mm petri dishes seeded with 750 μl OP50 in YT medium.

The fitness effects of mutations range continuously from lethal to deleterious to neutral to beneficial. In small populations, beneficial mutations can be lost and detrimental mutations fixed by random chance events (genetic drift). The loss or fixation of mutations depends upon both their selection coefficients (*s*) and the effective population size, *N*_*e*_. It has been shown that for sexually reproducing diploids, the dynamics of mutations with |s| << 1/2*N*_*e*_ are dominated by random genetic drift [73]. Therefore, small populations subjected to attenuated selection and an increased magnitude of genetic drift can potentially accumulate mutations with extremely large effects in addition to ones with moderate to very slight effects. With increasing population size, the efficiency of natural selection is increased. The differences in populations size in this MA experiment alters the relative importance of genetic drift versus natural selection in the fixation or loss of mutations, with genetic drift having the greatest influence in *N* = 1 lines and natural selection having greater influence in populations that were bottlenecked at 10 and 100 individuals each generation.

### RNA library preparation, sequencing and analysis of transcript abundance

The library preparation and RNA-sequencing procedures have previously been described in detail [72]. To prepare RNA from lines following 409 generations of mutation accumulation (all lines) or 25 or 100 generations (*N*.1 lines only), we isolated one, two and three individuals each of the *N*.1, *N*.10 and *N*.100 lines, respectively. These 55 worms, as well as one individual from the ancestral control population (preMA), were each sequestered to NGM plates seeded with OP50, where they were allowed to self-fertilize and reproduce at 20 °C. Three offspring worms at the L4 larval stage were isolated from each of the F1 populations to serve as biological replicates. These 168 individual worm samples were allowed to reproduce for three generations to yield enough tissue for RNA extraction. A standard bleaching protocol was used to collect eggs from gravid adults in order to generate synchronized populations of L1 larvae. Total RNA was isolated from L1 larvae via the Qiagen RNeasy Mini Kit. The Nanodrop 2000, Qubit 3.0 Fluorometer and an Agilent RNA Analyzer were used to evaluate the quality of the RNA samples, and an Illumina TruSeq RNA Library Prep Kit v2 was used with standard procedures to prepare the RNA sequencing libraries for each sample at the Texas A&M University Genomics and Bioinformatics Services Center. The RNA was fragmented and Illumina adapters were annealed for amplification. Size-selected cDNA fragments were isolated via a Qiagen Gel Extraction Kit. Finally, sequencing was performed on the Illumina HiSeq 4000 platform with default quality filters.

Demultiplexing and prefiltering of the sequencing reads was performed based on default Illumina QC protocols. Reads containing abnormally short insert lengths were removed, and adapters were discarded from the reads. The raw RNA-sequencing reads in fastq format were aligned to the protein-coding transcriptome of *C. elegans* (Wormbase reference N2 genome version WS247) using TopHat [74] via the “very sensitive” bowtie2 algorithm with a maximum of one mismatch in the anchor region for each spliced alignment and a minimum and maximum intron length of 20 and 3000 bp, respectively. Cufflinks [75] with default settings and gene annotations from the N2 genome version WS247 was used to estimate the relative transcript abundance for each protein-coding gene. All following analyses were focused on FPKM values calculated on the per gene level. The relative transcript abundances (FPKM) from the three biological replicates for each original sample were averaged to get mean relative transcript abundance for each gene in that sample.

### Small RNA sequencing

MA lines were synchronized using hypochlorite treatment and embryos were isolated after 12 h. RNA was extracted using TRIzol and small RNA libraries were prepared using the Illumina Small RNA sequencing kit as described previously [18]. Small RNAs were aligned to a genome built using bowtie from a fasta file containing all piRNAs, miRNAs, ncRNAs and genes including TEs, extracted from Wormbase (WS264; ce11), requiring perfect mapping. Reads mapping to the sense strand of ncRNAs and miRNAs were extracted using bedtools intersect –c –S. We used DEseq using ncRNAs and miRNAs to extract size factors. 22G-RNAs mapping to TEs and genes were extracted using a custom Perl script and the number of 22G-RNAs mapping antisense to each gene and TE was then counted using bedtools intersect –c –s. The 22G-RNAs were then normalized to the size factors from ncRNAs and miRNAs combined.

### TE copy-number analysis

DNA sequence reads were aligned to a genome built using bowtie2-build from TE consensus sequences extracted from repbase combined with all coding sequences. Bowtie2 was used to map PE reads to this genome and the read count mapping to each CDS or TE was obtained using bedtools intersect –c.

### Computational analysis of TE expression and small RNA analysis

TEs from WS264 were annotated using Repeatmasker [76]. All data analysis was conducted using the R environment for statistical analysis (www.Rproject.com). Details of the individual analyses are available on the Github page associated with this project (https://github.com/PeterSarkies-LMS/TEMALs_repo). Previously published datasets containing chromatin domain annotations from Early Embryo ChiP-Seq were taken from Evans et al. (2016) updated to WS264 using liftover (https://genome.ucsc.edu/). The average PATC score for each TE was calculated by taking the average PATC score across the element from per-base sliding window genome-wide PATC scores from [60]. To identify protein-coding genes near to TEs, we used the function windowbed from the Bedtools suite using a 2-kb window.

### Statistical analyses

To test if TE expression was significantly different across increasing population size, we used the Jonckheere test, a non-parametric test that takes account of a known ordering across the samples [77]. We implemented the Jonckheere test using the package clinfun in R. Additionally, to investigate whether the trend could be described linearly, we computed a standard linear model using the function lm in R. To compare if *N*.1 lines were different from all *N* > 1 lines, we used the Wilcoxon-unpaired test to avoid the assumption of normality. We calculated the correlation between 22G-RNA levels and expression of individual TEs using Spearman’s rank correlation coefficient, which does not assume linearity. Similarly, the correlation between TE expression changes and expression changes of nearby genes were obtained using Spearman’s rank correlation coefficient. Significance was defined at 0.05 after multiple test correction using the Benjamini-Hochberg procedure. To estimate the number of significant correlations between TE and nearby genes that might be expected by chance, we replaced each protein-coding gene with a random protein-coding gene greater than 2 kb distant. We then calculated the correlation coefficients for this random set and tabulated the number of significant correlations. We repeated this process 100 times to obtain a simulated distribution and compared this to the observed number of significant correlations.

## Supplementary information

Additional file 1: **Figure S1.** Quantitative analysis of TE variability across different population sizes. A Mean change in expression relative to the ancestral control across all TEs for each line. B Violin plots showing distribution of changes in TE expression across all TEs for each line. Lines are ordered by increasing mean TE expression change (L to R). C Fano factor of individual TE transcript levels across all lines of the indicated population size. D Fano factor in individual protein-coding gene transcript levels across all lines of the indicated population size. E Fano factor of TE transcript levels in 1000 samples of five *N*.1 lines, 253 samples of five *N*.10 lines [the maximum] and all five *N*.100 lines. F Total variance in transcript level differences between each TE and its corresponding value in the starting population across lines of different population size. G Total variance of transcript level differences between each protein-coding gene and the starting population in lines of the indicated population size. Boxplots for A-D are as in Fig. 1D. H Variance in TE transcript changes relative to starting population compared to the variance in protein-coding gene transcript changes in the same line. **Figure S2.** Characteristics of *C. elegans* TEs analysed in this study. A Base pairs covered by TE families for which robust RNA-Seq coverage was obtained in at least one line (analysed in this study), grouped by family and coloured according to type of TE. B Number of elements of each TE family found, coloured according to type of TE. C Distribution of TEs analysed in this study across chromosomes. D Number of TEs analysed in this study targeted by different small RNA pathways. E Number of TEs analysed in this study in different chromatin domains. **Figure S3.** Further analysis of small RNA mediated TE control in MA lines. A Number of piRNA loci containing a mismatch to the genome of the parental line across different population sizes of MA lines, as assessed by small RNA sequencing. B Total piRNA expression across lines of different population sizes. C 22G-RNAs targeted to TEs targeted by piRNAs across different population sizes. D Scatterplot comparing 22G-RNA levels and mRNA levels for Turmoil2 elements across all lines. E Spearman’s rank correlation coefficient between expression changes and 22G-RNA levels for different TEs across all lines, coloured by HRDE-1 targets. F Spearman’s rank correlation coefficient between expression changes and 22G-RNA levels for different TEs across all lines, coloured by WAGO-1 targets. G Total expression changes of TEs targeted by HRDE-1 across different population sizes. H Total expression changes of TEs targeted by WAGO-1 across different population sizes. **Figure S4.** TEs on the X chromosome are not upregulated in MA lines. A Expression of TEs located on autosomes across different population sizes. B Expression of TEs located on the X chromosome across different population sizes. See Additional File 1: Fig. S2 for the numbers of TEs on each chromosome. **Figure S5.** Further analysis of the effect of sequence features on TE desilencing in MA lines. A GC-content does not affect reactivation of TEs in the *N*.1 lines. Bins with high to low GC-content, left to right. There are no significant (q < 0.1) trends in any category. Regression lines were therefore omitted for clarity. B GC-content does not affect the change in 22G-RNA levels. Bins with high to low-GC content, left to right. There are no significant trends in any category so regression lines were omitted as in A. C No clear difference in the proportion of TEs from different chromatin domains within bins of different PATC content. D Expression of TEs from regulated, heterochromatic and active chromatin regions in the top PATC bin. E Expression of TEs from regulated, heterochromatic and active chromatin regions in the lowest PATC bin. (PDF 2643 kb)

Additional file 2. Table showing change in median expression of individual transposable elements in MA lines. (CSV 8 kb)

## Data Availability

RNA sequencing for *N.*1, *N.*10 and *N.*100 lines after 409 generations is available from GEO GSE112821 [72]; RNA sequencing for *N*.1 lines after 25 and 100 generations is available from the SRA PRJNA553063 [78]; DNA sequencing for *N*.1, *N*.10 and *N*.100 lines after 409 generations is available from SRA PRJNA448413 [72]; small RNA sequencing of *N*.1, *N*.10 and *N*.100 lines generated in this study is available from the SRA PRJNA656266; and *C. elegans* lines are available from V. Katju upon request. Lines will also be available in the CGC public repository once deposition becomes possible. R code used to generate the figures in this manuscript along with processed files used to generate the figures have been uploaded to a GitHub repository https://github.com/PeterSarkies-LMS/TEMALs_repo
