# Supplementary figures and images for: Long-term experimental evolution reveals purifying selection on piRNA-mediated control of transposable element expression
Source: BMC Biol. 2020 Nov 6;18:162. doi: 10.1186/s12915-020-00897-y (PMC7646084; doi:10.1186/s12915-020-00897-y)

Figure S1

A

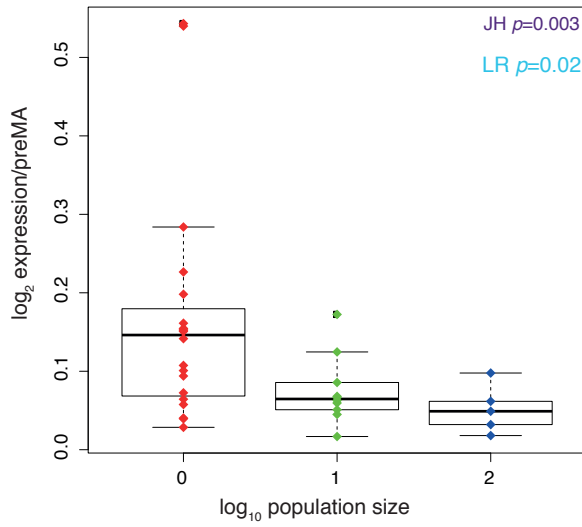

B

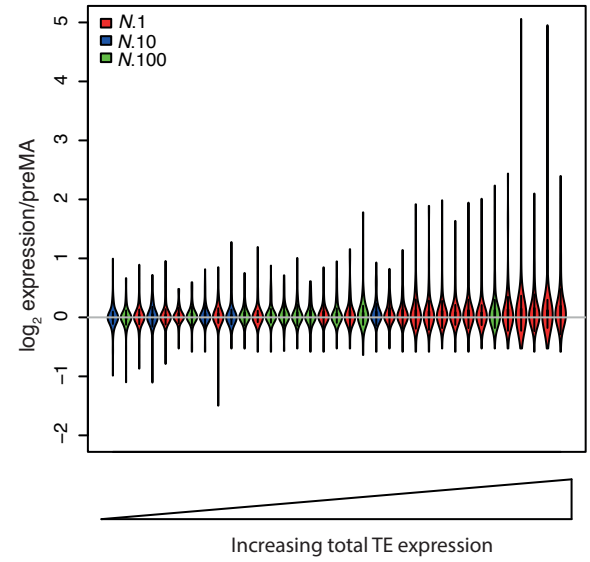

C

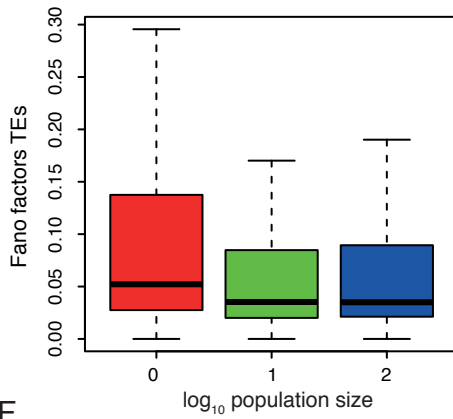

D

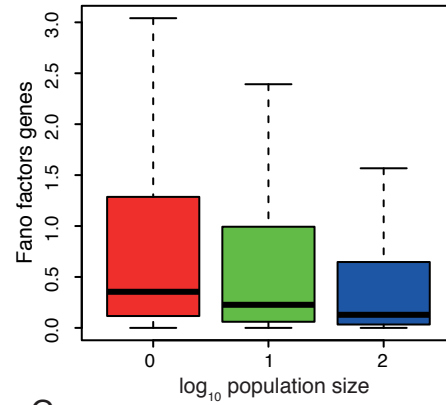

E

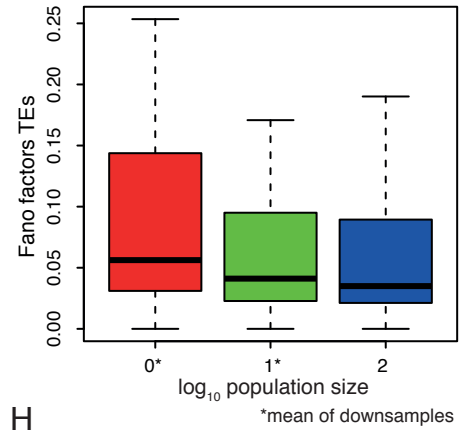

F

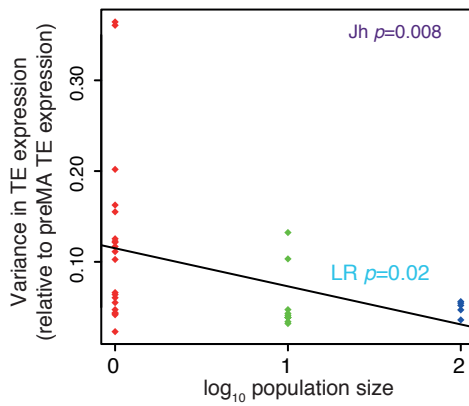

G

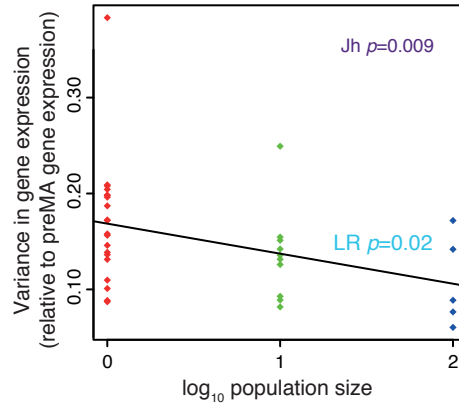

H

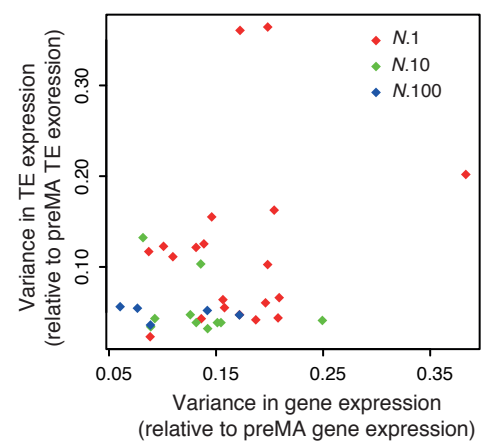

Figure S2

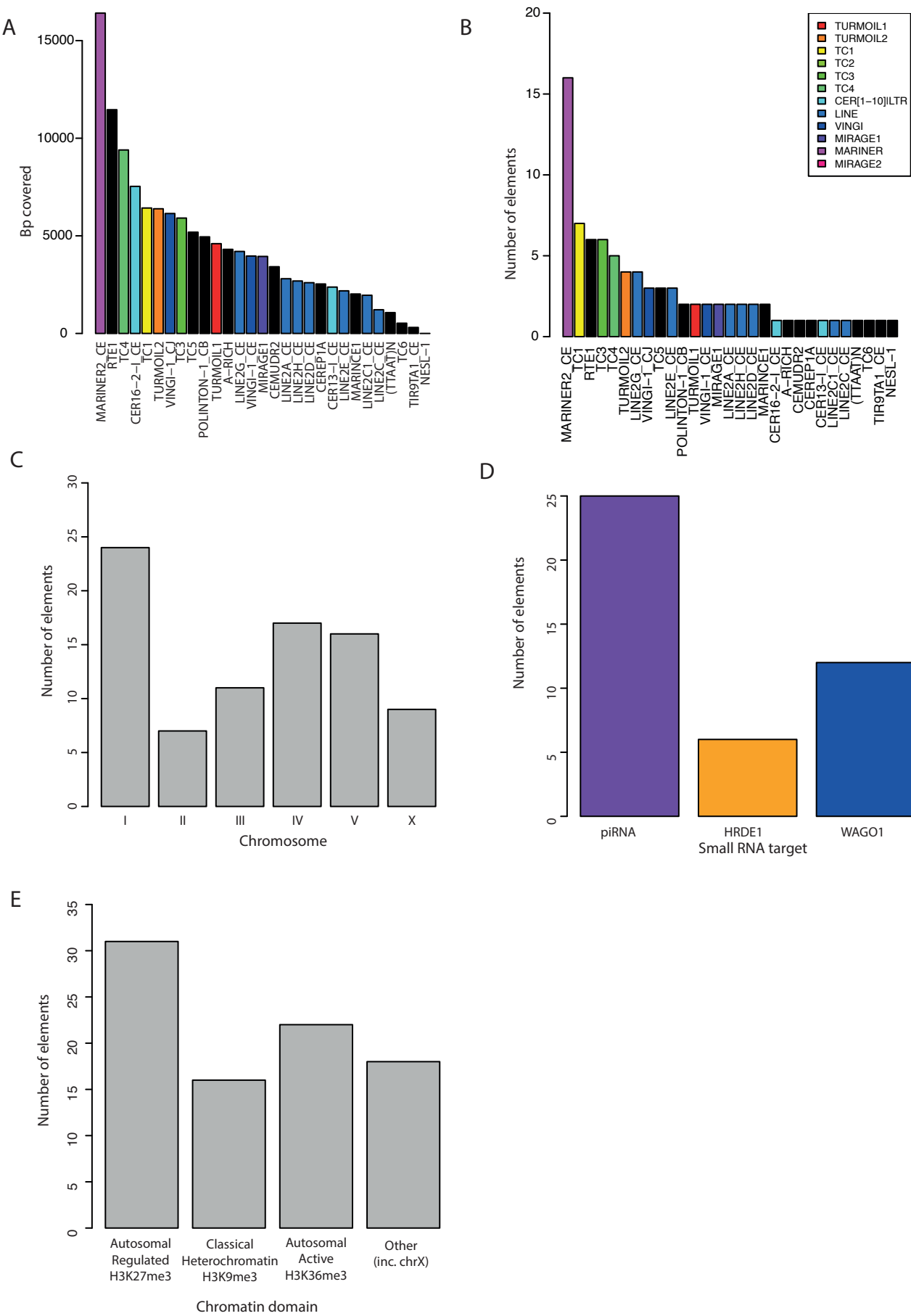

# Figure S3

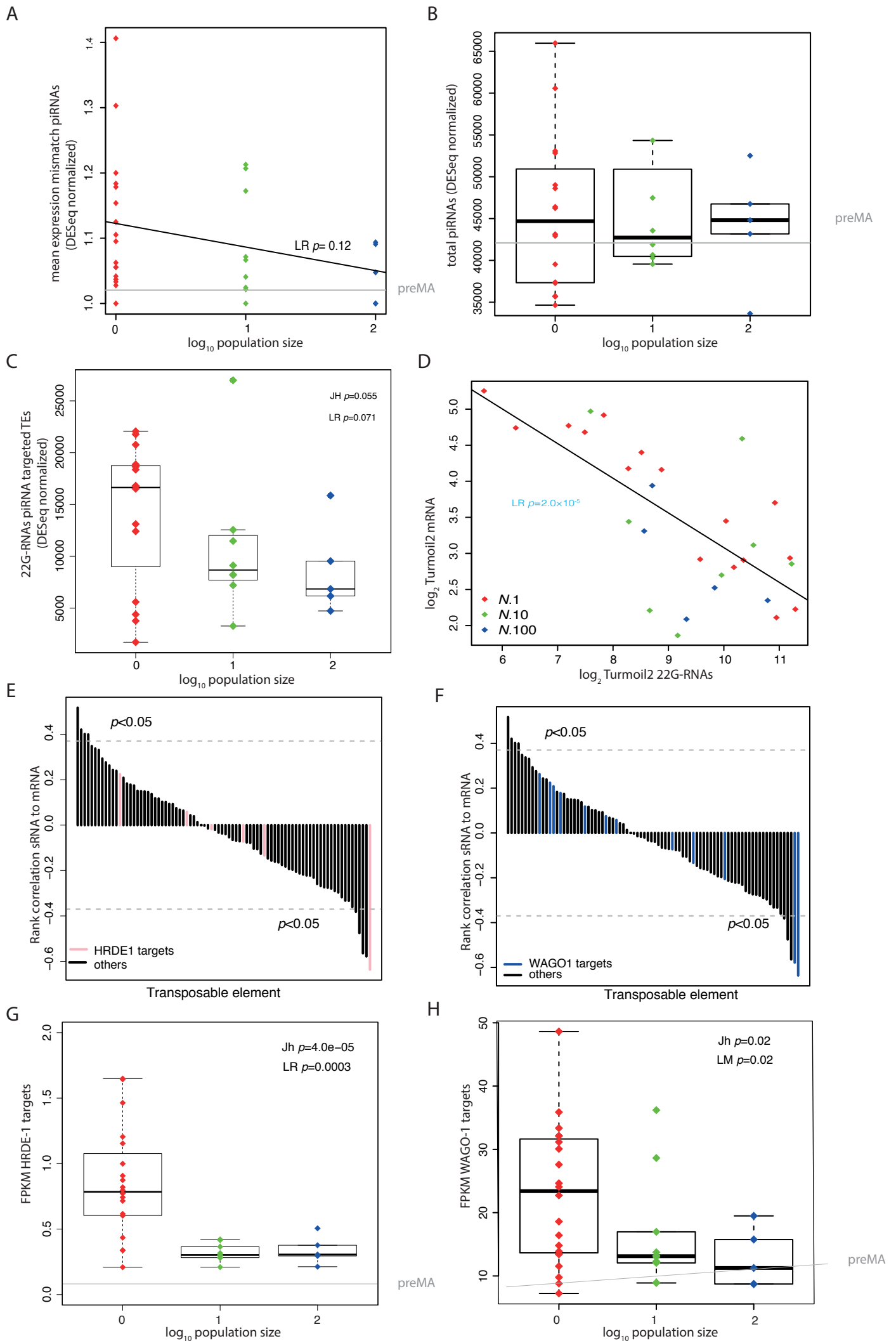

Figure S4

A

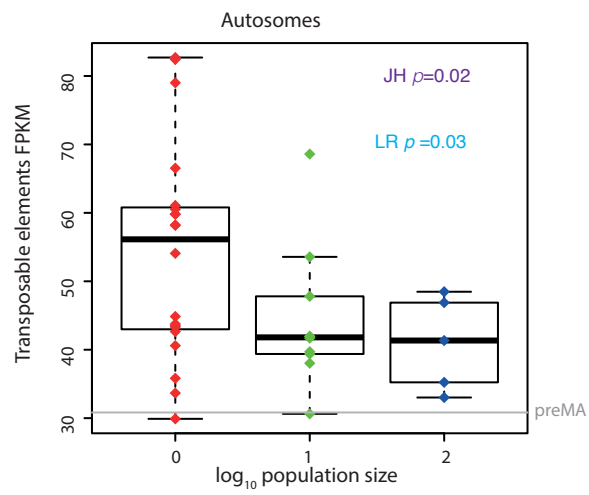

B

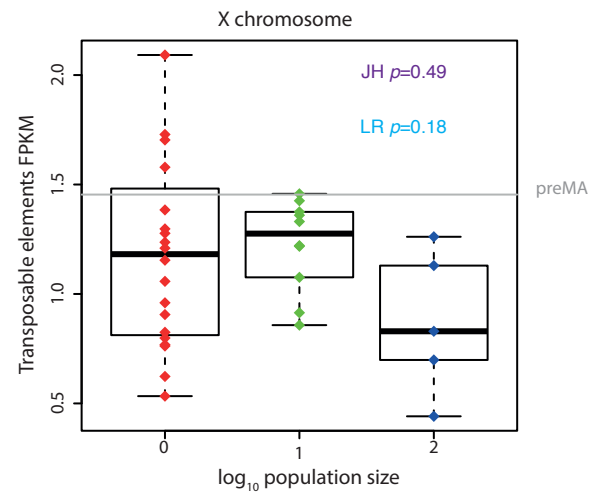

Figure S5

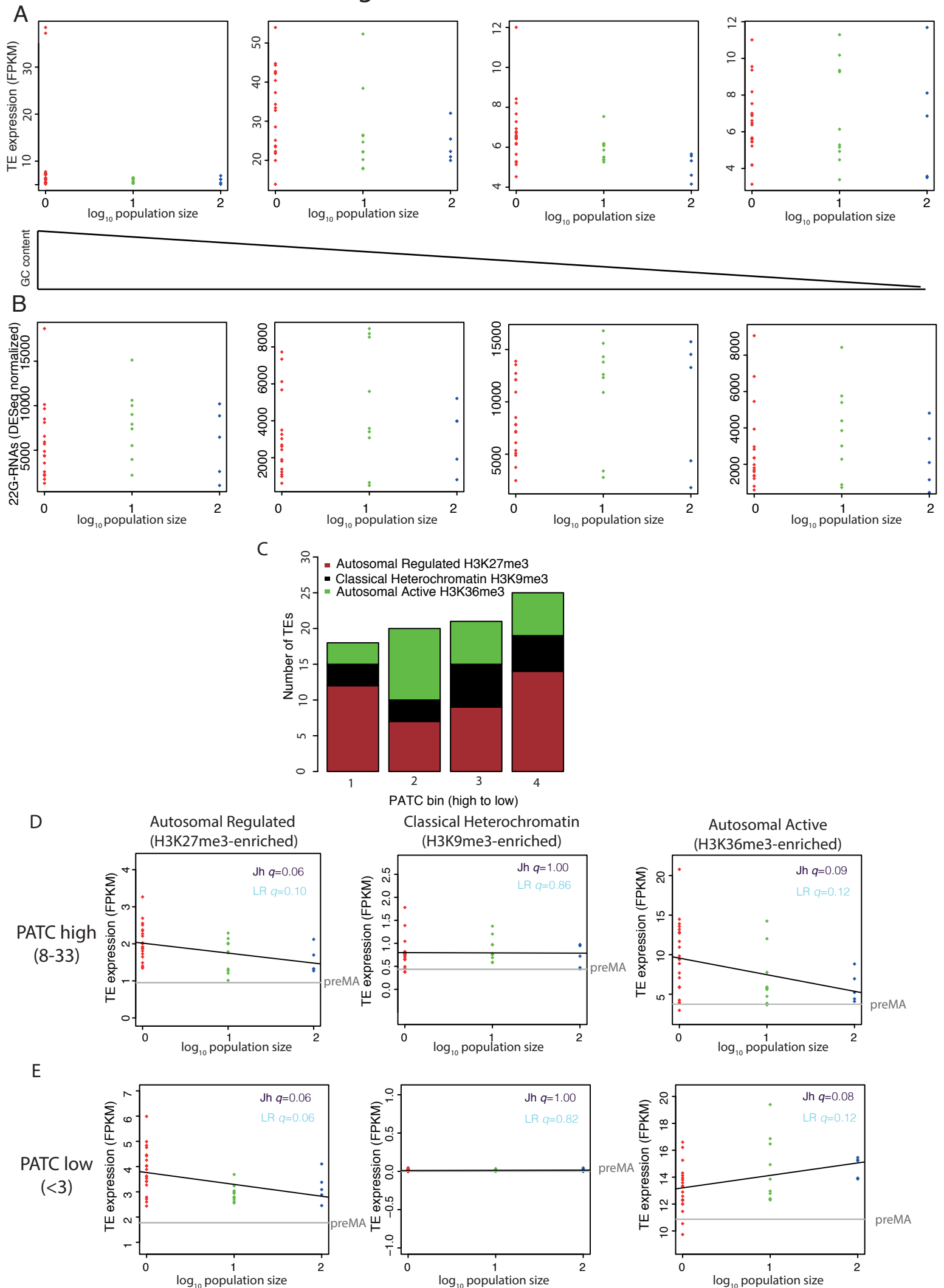

Supplement: Supplementary file 1 — Figure S1. Quantitative analysis of TE variability across different population sizes. A Mean change in expression relative to the ancestral control across all TEs for each line. B Violin plots showing distribution of changes in TE expression across all TEs for each line. Lines are ordered by increasing mean TE expression change (L to R). C Fano factor of individual TE transcript levels across all lines of the indicated population size. D Fano factor in individual protein-coding gene transcript levels across all lines of the indicated population size. E Fano factor of TE transcript levels in 1000 samples of five N.1 lines, 253 samples of five N.10 lines [the maximum] and all five N.100 lines. F Total variance in transcript level differences between each TE and its corresponding value in the starting population across lines of different population size. G Total variance of transcript level differences between each protein-coding gene and the starting population in lines of the indicated population size. Boxplots for A-D are as in Fig. 1D. H Variance in TE transcript changes relative to starting population compared to the variance in protein-coding gene transcript changes in the same line. Figure S2. Characteristics of C. elegans TEs analysed in this study. A Base pairs covered by TE families for which robust RNA-Seq coverage was obtained in at least one line (analysed in this study), grouped by family and coloured according to type of TE. B Number of elements of each TE family found, coloured according to type of TE. C Distribution of TEs analysed in this study across chromosomes. D Number of TEs analysed in this study targeted by different small RNA pathways. E Number of TEs analysed in this study in different chromatin domains. Figure S3. Further analysis of small RNA mediated TE control in MA lines. A Number of piRNA loci containing a mismatch to the genome of the parental line across different population sizes of MA lines, as assessed by small RNA sequencing. B To [file 12915_2020_897_MOESM1_ESM.pdf]
